# Determination of a New Parameter, Elevated Epiretinal Membrane, by En Face OCT as a Prognostic Factor for Pars Plana Vitrectomy and Safer Epiretinal Membrane Peeling

**DOI:** 10.1155/2015/838646

**Published:** 2015-10-04

**Authors:** Mitrofanis Pavlidis, Ilias Georgalas, Norbert Körber

**Affiliations:** ^1^Augencentrum Köln, Josefstraße 14, 51143 Cologne, Germany; ^2^Ophthalmology Department, University of Athens, Mesogeion 154, Athens, Greece

## Abstract

*Purpose*. To evaluate the significance of the area of epiretinal membrane (EM) that is not in contact with the retinal structure as a preoperative parameter for safer grasping of the EM and a prognostic factor for visual improvement/outcome. *Methods*. This prospective observational study included 73 consecutive patients (80 eyes) who underwent pars plana vitrectomy (PPV) and EM peeling. Corrected distance visual acuity (CDVA) and central foveal thickness (CFT) were evaluated preoperatively and at 12 months postoperatively. The number of initial peeling grasps was recorded in the operation protocol. The elevated EM portion was identified by en face optical coherence tomography (OCT) and processed digitally to calculate its area. *Results*. Surgery was found to significantly improve CDVA and decrease CFT. CDVA improvement correlated with elevated EM, preoperative CFT, and the number of grasping attempts. *Conclusion*. The detection of elevated EM via en face OCT could assist safer grasping of the EM and indicate the potential for visual outcome improvement after PPV and EM peeling.

## 1. Introduction

Epiretinal membrane (EM) is a translucent or semitranslucent fibrocellular proliferation formed on the internal surface of the retina [1]. Specifically, the development of EM involves glial cells, retinal pigment epithelial (RPE) cells, macrophages, fibrocytes, and collagen fibers [2, 3]. EM can be associated with posterior vitreous detachment, retinal breaks, vascular retinopathy, ocular inflammation, congenital ocular disorders, laser photocoagulation, or retinal detachment surgery [2]. However, membranes can also be idiopathic and have a benign evolution with few associated symptoms [4, 5]. EMs can contract, generating different levels of metamorphopsia, retinal edema, and degeneration of the underlying retina [4, 5].

Pars plana vitrectomy (PPV) combined with peeling of the EM, facilitated by use of a coloring agent, in conjunction with the rhexis of the internal limiting membrane (ILM), is the primary surgical option for treatment of EM [6–8], although surgical management is not always the most adequate option [9]. Various factors affect the outcome of EM surgery, such as preoperative visual acuity, duration of symptoms, presence of vitreoretinal traction, level of metamorphopsia, thickness of the EM, and presence of preoperative macular edema [10–14]. Analysis of the affected area by optical coherence tomography (OCT) has also been found to be useful in predicting the outcome of EM surgery [10, 15–17]. Shiono et al. [15] found that photoreceptor outer segment (PROS) length obtained by spectral domain-optical coherence tomography (SD-OCT) was a predictor of postoperative best corrected distance visual acuity (CDVA) in patients with idiopathic EM. Likewise, Odrobina et al. [10] suggested that vitreous surface adhesion might be a prognostic factor for the natural course of vitreomacular tractions. In two studies, Kim et al. [13, 18] conducted retrospective and prospective observational case series, which showed that the extent of EM-retinal adhesion and the presence of fibrillary changes determined by SD-OCT provides reliable preoperative assessment of surgical difficulty and helps localize surgically advantageous coordinates for the initiation of EM removal.

Since it is important to identify areas of elevated membrane for easier and safer initial grasping of the EM while avoiding touching and damaging the retina, our hypothesis was that analysis of this factor may be helpful in predicting surgical outcomes. The aim of the current study was to determine the significance of the area of EM that is elevated and not in contact with the retinal structure and central foveal thickness (CFT) as prognostic factors for EM surgery.

## 2. Methods

### 2.1. Patients

In this prospective consecutive case series study, 80 eyes of 73 patients aged between 58 and 82 years were analyzed. The inclusion criteria were patients with EM that was visualized easily upon fundoscopic and OCT examination (as a hyperreflective line partially in contact with the retinal surface associated with folds in the interior layers of the retina, retinal thickening, and distortion of the normal retinal architecture) and a deterioration of visual acuity with or without metamorphopsia. EM was graded according to the criteria of Klein and coauthors [19]. The exclusion criteria were the presence of EM for longer than 2 years, phakic patients, previous PPV for EM, EM due to diabetic maculopathy, EM due to retinal vessel occlusions, glaucoma, ocular hypertension, penetrating ocular trauma, central serous retinopathy, subfoveal drusen, solar maculopathy, ocular inflammation, ocular ischemia, corneal disease, prolonged use of topical steroid medication, and the use of medications that are potentially related to the development of macular cysts (e.g., systemic niacin and topical prostaglandin analogues). All subjects were adequately informed about standard PPV for EM using the elevated membrane OCT report and signed a consent form. The study adhered to the tenets of the Declaration of Helsinki, and local regulatory requirements were fulfilled.

### 2.2. Study Protocol

Preoperatively, all patients received a full ophthalmologic examination including refractive status, uncorrected visual acuity, CDVA (decimal scale), slit-lamp examination, Goldmann applanation tonometry, and OCT examination (Cirrus HD-OCT 4000; Carl Zeiss Meditec, Dublin, CA, USA) incorporating CFT analysis and fundoscopy. Postoperatively, patients were evaluated at 1 day, 1 week, and 3, 6, and 12 months after surgery. At 1 day after surgery, only uncorrected distance vision, intraocular pressure, and the integrity of the retinal structure as assessed by OCT were evaluated. All complications were noted during follow-up examinations.

### 2.3. Imaging of the Elevated EM

**Figure 1:**
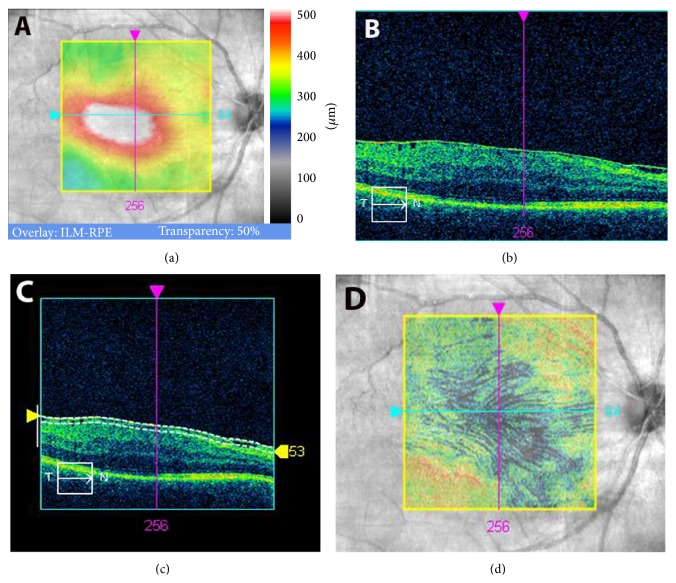
Diffuse infiltrating epiretinal membrane (EM) with a small elevated area. (a) Macular thickness map obtained with spectral domain-optical coherence tomography (SD-OCT). (b) Line scanning ophthalmoscope (LSO) SD-OCT image. (c) Automatic identification of the EM surface (upper white line) and selection of the retinal surface (lower white line). (d) Black pixelated areas calculated automatically reveal elevated EM areas, overlaid with the LSO image in transparency.

**Figure 2:**
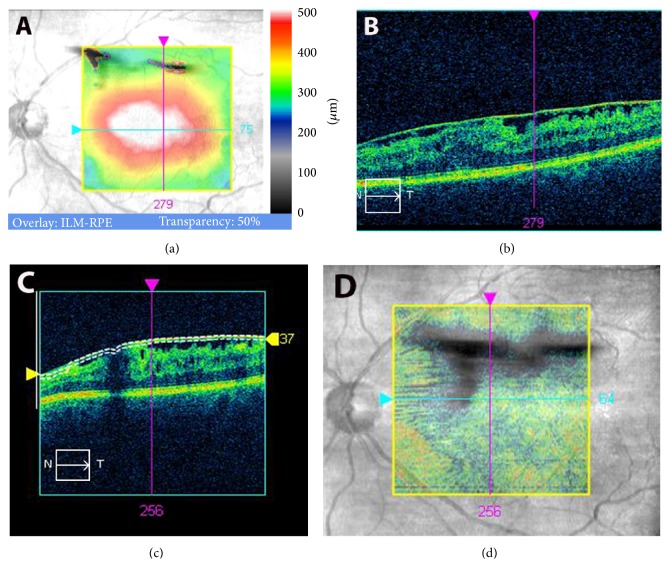
Large area of elevated epiretinal membrane (EM). (a) Macular thickness map obtained with spectral domain-optical coherence tomography (SD-OCT). (b) Line scanning ophthalmoscope (LSO) SD-OCT image. (c) Selection of the slab layer of the internal limiting membrane (ILM). (d) ILM overlay selection over LSO image in transparency reveals elevated part of EM. Large darker areas of elevated EM indicate potential points for safer initial grasping of the membrane.

**Figure 3:**
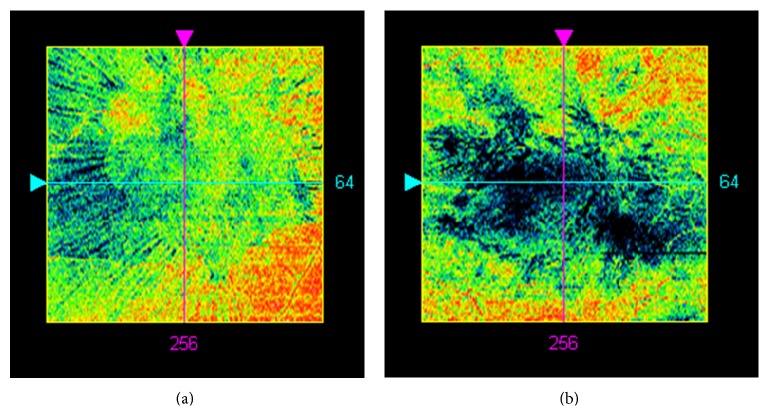
Two epiretinal membrane- (EM-) infiltration examples using the en face calculated elevated membrane report. The black areas represent the EM areas with no retinal contact (small and large areas in (a) and (b), resp.).

At the final preoperative visit, line scanning ophthalmoscope (LSO) fundus images with an ILM slab overlay were obtained by performing macular cube 512 × 128 scans with Cirrus HD-OCT system. Cases with artifacts in the OCT cube mode were excluded from the study. En face OCT images of the EM were used as follows. In the advanced visualization mode, the ILM slab was selected. Cirrus HD-OCT identifies automatically the surface of the EM-retinal complex as a line (Figures 1(c) and 2(c), upper white line). A second lower white line can be moved to fit to the lowest point on the vitreoretinal interface. The distance between the two lines depends on the elevation of the EM from the retina and was found to be on average 45 ± 8 *μ*m. Furthermore, in the advanced visualization mode, selection of the ILM slab projected automatically the black pixelated area of the elevated membrane onto the LSO fundus image of the macula, with a transparency of 70%, for simultaneous identification of the elevated EM and retina (Figures 1(d) and 2(d)). The results obtained were recorded as advanced visualization custom reports and exported in.jpg format (Figures 1 and 2). These reports were used during surgery to assist the surgeon when choosing an elevated portion of EM to grasp for initial membrane peeling. The images were then analyzed later with conventional software (Photoshop 10.0) by means of pixel measurement (Figure 3).

### 2.4. Surgery

All vitreoretinal surgeries were performed by the same experienced surgeon (MP) who used the standard 3-port 27-gauge PPV technique (EVA DORC, Netherlands). The number of protocol-documented initial retinal grasps during EM peeling was statistically evaluated in order to determine its correlation with CDVA. Initial grasps were defined as forceps grasps which initiated separation of the membrane from the retina. These were orientated to the largest area of elevated EM by observation of the black pixelated area on the printed report (Figures 1(d) and 2(d)). Subsequent forceps grasps targeted the already elevated edges of EM without further retinal contact. Further forceps activities needed for complete removal of the already separated EM were excluded from the evaluation.

Visualization of the fundus was achieved using a Resight 500 viewing system with a 90 dpt lens (Carl Zeiss Meditec). Brilliant Blue G- (BBG-) assisted EM peeling was performed using end-gripping forceps (Eckardt ILM-DORC), which was followed by the remains of the ILM identified by restaining of the retina with BBG and secondary peeling. BBG has been used for labeling previously as it has less toxic effects on the macula [20]. Postoperatively, prophylactic treatment consisting of anti-inflammatory and antibiotic drops was prescribed for 1 month.

### 2.5. Statistical Analysis

SPSS statistics software package version 19.0 for Windows (IBM, Armonk, NY, USA) was used for statistical analysis. The normality of all of the data samples was first evaluated by the Kolmogorov-Smirnov test. When parametric analysis was possible, Student's *t*-test for paired data was performed for all parametric comparisons between preoperative and postoperative examinations. When parametric analysis was not possible, Wilcoxon test or Bortz and Schuster test was applied to assess the significance of differences between preoperative and postoperative data, using the same level of significance (*p* < 0.05) in all cases. Correlation coefficients (Pearson or Spearman, depending on whether the normality condition could be assumed) were used to assess the correlation between different variables.

## 3. Results

The mean age of the patients enrolled in this study was 70 years (standard deviation [SD]: 20; range: 58 to 84 years). According to the EM grading system of Klein et al. [19], EM grades 1, 2, and 3 were present in 3 eyes (3.75%), 65 eyes (81.2%), and 12 eyes (15.0%) of the analyzed sample, respectively.

### 3.1. Visual Outcomes

After surgery, CDVA after 12 months improved significantly (*p* < 0.01, Wilcoxon test) from a mean preoperative value of 0.44 (SD: 0.17; median: 0.40; range: 0.10 to 0.80) to a mean postoperative value of 0.72 (SD: 0.23; median: 0.80; range: 0.00 to 0.70). Therefore, the mean change in CDVA was 0.27 (SD: 0.19; median: 0.20; range: 0 to 0.90). No losses in CDVA were detected in any of the evaluated samples.

### 3.2. OCT Outcomes

After surgery, CFT after 12 months decreased significantly (*p* < 0.01, Wilcoxon test) from a mean preoperative value of 439.11 *μ*m (SD: 85.4; median: 424.5; range: 0 to 668.0 *μ*m) to a mean postoperative value of 374.2 *μ*m (SD: 58.71; median: 362.5; range: 280.0 to 600.0 *μ*m). Therefore, the mean CFT change after surgery was −64.9 *μ*m (SD: 60.9; median: −51.5; range: −256.0 to 11.0 *μ*m).

Regarding the preoperative EM area with no retinal contact, a mean value of 40958.47 pixels was found according to the procedure described in this study (SD: 5010.14; median: 39721.0; range: 34163.0 to 51595.0 pixels).

### 3.3. Correlation between Visual Acuity and OCT Outcomes

**Figure 4:**
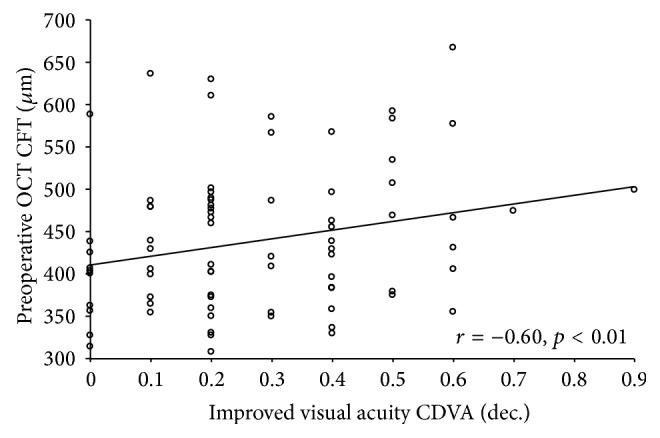
Scattergram showing the relationship between the change in corrected distance visual acuity (CDVA) and preoperative central foveal thickness (CFT). The line of best fit obtained by the least-squares fitting method is shown.

A statistically significant correlation was found between the change in CDVA and preoperative CFT (*r* = −0.6, *p* < 0.01) (Figure 4). CDVA change and CFT were correlated according to the following formula: change  in  CDVA = −0.001 × CFT_preop_ + 0.431 (*R* ^2^: 0.36).

**Figure 5:**
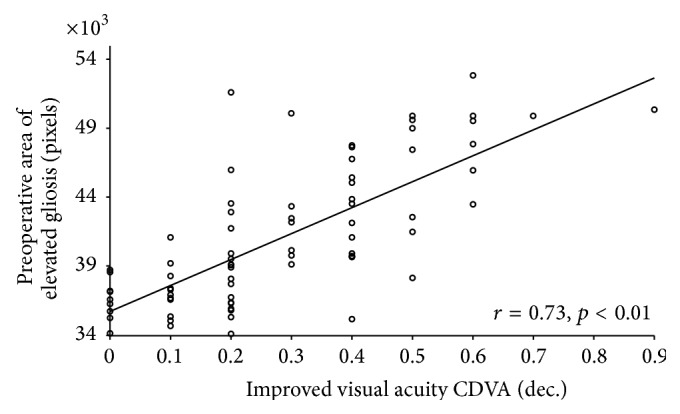
Scattergram showing the relationship between the change in corrected distance visual acuity (CDVA) and preoperative epiretinal membrane (EM) area (pixels) with no retinal contact (elevated EM). The line of best fit obtained by the least-squares fitting method is shown.

**Figure 6:**
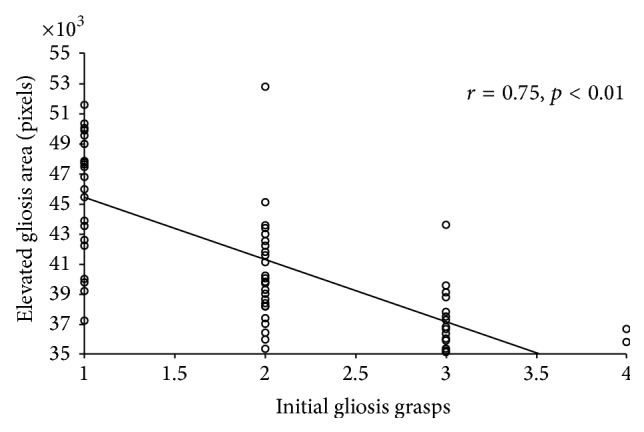
Scattergram showing the relationship between the number of initial grasps used for epiretinal membrane (EM) peeling and the preoperative EM area (pixels) with no retinal contact (elevated EM). The line of best fit obtained by the least-squares fitting method is shown.

The preoperative EM area with no retinal contact correlated significantly with CDVA improvement (*r* = 0.73, *p* < 0.01) (Figure 5) and with the number of documented intraoperative initial peeling grasps (*r* = −0.75, *p* < 0.01) (Figure 6).

### 3.4. Comparison of Two Cohorts Depending on the Area of Elevated EM

Differentiation of smaller areas (33849 to 39702 pixels) and larger areas (39740 to 52816 pixels) of elevated EM revealed a significant correlation of CDVA improvement with the larger area cohort. In the smaller area cohort, the mean value of CDVA improvement after 12 months of follow-up was 0.14 (SD: 0.12). In the larger area cohort, the mean CDVA improvement after 12 months of follow-up was 0.41 (SD: 0.16) (*p* < 0.01).

### 3.5. Comparison of Two Cohorts Depending on the Baseline CDVA

Differentiation of lower baseline CDVA values (0.05 to 0.3) and higher baseline CDVA values (0.4 to 0.8) indicated a smooth but not significant improvement of CDVA in the cohort with lower baseline CDVA. The mean improvement in the lower baseline CDVA cohort was 0.3 (SD: 0.2). The mean improvement in the higher baseline CDVA cohort was 0.2 (SD: 0.1) (*p* > 0.05).

### 3.6. Comparison of Two Cohorts Depending on the Baseline Foveal Contact of the EM

Differentiation of an on group (EM with foveal contact, *n* = 54) and an off group (fovea with elevated EM, *n* = 26) indicated a significantly higher improvement of CDVA in the off group (on group, mean: 0.15; SD: 0.11 and off group, mean: 0.52; SD: 0.13).

## 4. Discussion

EM peeling is a difficult and risky surgical procedure. Initial grasping of the EM is difficult because of the risk of grasping the retina under the membrane and consequently causing irreversible trauma to the underlying ganglion cells and axons. Elevated areas of EM are easier to grasp, and their removal is safer because retinal contact is avoided during surgical manipulation (Figure 1). For this reason, we thought that OCT analysis of elevated areas of EM might assist with the initial grasping of EM and potentially predict visual outcomes after surgery. Likewise, the value of CFT as a predictor of visual outcomes after EM surgery was also evaluated.

In the analyzed sample, a statistically significant mean improvement in CDVA of 3 decimal increments was achieved. This is consistent with the previous results of several authors who have reported the visual benefits of PPV and peeling in EM [7, 8, 10, 11, 21–27]. Indeed, a significant reduction of CFT was observed in our series as has been reported in other studies evaluating macular structural changes by OCT [21, 23, 26]. In a comparative study of eyes undergoing EM surgery with and without ILM peeling, Kwok et al. [8] found that the removal of ILM was able to minimize the recurrence of EM, without adverse visual outcomes. In the current study, CFT was significantly correlated with visual changes after surgery. Specifically, more visual improvement was achieved in those cases with a higher CFT or more macular edema. Therefore, those cases with more macular traction leading to a significant increase in macular thickness may show higher visual gain with surgical removal of EM tractions. By conducting a retrospective observational study of cases of vitreomacular traction, Odrobina et al. [10] suggested that it might be advantageous to perform PPV along with ILM peeling only in eyes with broad vitreous adhesions or coexisting EM. Falkner-Radler et al. [12] conducted a prospective study to evaluate certain OCT parameters in EM and concluded that analysis of the ILM profile and foveal contour may help in understanding several visual outcomes after surgery. Likewise, Kim et al. [13] found a significant correlation between CDVA after EM surgery and early postoperative CFT. Recent studies have found that the presence of an intact inner/outer segment (IS/OS) junction on the preoperative SD-OCT scan was an important predictor of better visual recovery after EM surgery [28, 29]. Macular edema and IS/OS junctions are associated with long-term disease and thus increased damage to photoreceptor cells and worsened functionality. Further study is needed to analyze possible correlations between intraretinal junctions, CDVA changes, and elevated EM.

The present study shows that visual improvement is significantly correlated with the preoperative area of elevated EM, as measured by OCT; this new information may be used to determine the prognosis of EM surgery. EM that infiltrates the retina appears to cause more tissue damage than elevated EM does. Kim et al. [18] and Seidel et al. [17] found that there is a correlation between high infiltrated membranes, remaining ILM after peeling, and visual outcome. This may be because EM with a large area of retinal infiltration induces more retinal tissue damage over the period of its development or because the EM peeling procedure itself is more traumatic in cases of EM with a large area of retinal infiltration than in cases of EM with few retinal attachments.

In our study, we also focused on the fovea, which is responsible for CDVA, and found a significantly higher improvement of CDVA in the group with EM elevated off the fovea than in the group with EM-foveal contact. The influence of the area of elevated EM on the number of initial forceps grasps as well as visual outcome after PPV and EM peeling was also highly significant; surgeons could potentially use this information to conduct a safer grasping procedure by initiating peeling at elevated areas of EM.

In conclusion, PPV combined with EM peeling, facilitated by a coloring agent, is an effective procedure that can reduce the CFT and improve visual acuity. More visual benefits are expected in EM eyes with larger areas of elevated membrane, in eyes with more significant macular edema, and, potentially, in eyes with more significant tractions. This study shows that en face OCT can be used to recognize elevated areas of EM, which could be useful for reducing initial EM grasping and as a predictive factor for visual outcomes after PPV and EM peeling.

## Supplementary Material

This video demonstrate the projection of the elevated membran areas (black pixels) on the retina image coming from the microscope. Based on this navigation membran peeling can be then initiated on the elevated membran areas without traumatising the retina.

## Summary Statement

En face optical coherence tomography (OCT) identification of areas with elevated epiretinal membrane (EM) could be useful for safer grasping of the EM via gripping of the elevated parts. A better visual outcome is expected after pars plana vitrectomy and EM peeling in eyes with larger areas of elevated EM.
